# Contralateral tension pneumothorax after extubation following thoracoscopic wedge resection: a case report

**DOI:** 10.3389/fmed.2026.1958948

**Published:** 2026-10-26

**Authors:** Sijie Chen, Na Zhou, Yiting Zhu, Xiaohui Yang, Huifen Lin

**Affiliations:** Nursing Department, Sir Run Run Shaw Hospital, Zhejiang University School of Medicine, Hangzhou, China

**Keywords:** case report, post-anesthesia care unit, postoperative complications, tension pneumothorax, thoracic surgery

## Abstract

Contralateral tension pneumothorax is a rare but potentially life-threatening complication after thoracic surgery and may be difficult to distinguish from more common postoperative respiratory complications. We report a case of a 53-year-old female patient who developed a right tension pneumothorax during recovery from anesthesia following a thoracoscopic left wedge resection performed under one-lung ventilation. The patient experienced severe coughing and choking during extubation; immediately after extubation, she initially presented with dyspnea and bilateral wheezing, raising suspicion of bronchospasm. Despite aggressive diagnostic and therapeutic efforts, respiratory abnormalities persisted, and the patient was ultimately diagnosed with a right-sided tension pneumothorax. Following emergency needle decompression, surgical exploration was immediately performed, revealing a ruptured band of right-sided pleural adhesions accompanied by ongoing air leakage. After definitive treatment, the patient made a smooth recovery. This case highlights that when unexplained respiratory abnormalities occur following thoracic surgery, even if initial oxygenation is adequate, the possibility of contralateral tension pneumothorax should be considered, and cough management should be optimized.

## 1 Introduction

Lung isolation with a double-lumen tube (DLT) is commonly used during lobectomy to facilitate one-lung ventilation. However, the relatively large and rigid DLT may increase airway irritation and provoke vigorous coughing during extubation (1, 2), resulting in a transient increase in intrathoracic pressure (3). This may cause lung injury and pneumothorax on the operative side and, in rare cases, affect the contralateral lung (4, 5). When air enters the contralateral pleural space, a contralateral pneumothorax develops; if the resulting pressure compromises the mediastinum, tension pneumothorax (TP) may occur. Without prompt diagnosis and treatment, TP can lead to severe hypoxemia, hypotension, and even cardiac arrest (6). Early manifestations of contralateral TP are often nonspecific and may mimic more common perioperative complications, such as atelectasis, bronchospasm, and mucus plugging, particularly during early recovery in the post-anesthesia care unit (PACU) (7, 8). Here, we report a case of right-sided TP triggered by vigorous coughing during early recovery after left lung wedge resection. The timing and clinical manifestations overlapped with common perioperative respiratory and cardiovascular abnormalities, complicating early diagnosis. This case highlights the importance of considering contralateral TP after thoracic surgery and managing coughing during extubation.

## 2 Case presentation

A 53-year-old woman (165 cm, 67 kg, BMI 24.6 kg/m^2^) was admitted for surgical treatment of a pulmonary nodule. She had no history of smoking or chronic disease. Preoperative airway assessment revealed Mallampati Class III, limited head and neck mobility, and a thyromental distance of 5 cm. Her family also reported habitual snoring. Arterial blood gas analysis on room air showed partial pressure of oxygen (PaO₂) 77 mmHg and partial pressure of carbon dioxide (PaCO₂) 42 mmHg. Preoperative Chest computed tomography (CT) revealed mild bilateral pleural thickening and scattered fibrotic changes (Figure 1A). The upper pleural layers were slightly thickened. Pulmonary function tests showed normal ventilatory function：FEV₁/FVC = 83%(ratio of forced expiratory volume in one second to forced vital capacity). Bronchoscopy revealed no neoplasms or active bleeding in the bilateral bronchi, but showed inflammation of the bronchial mucosa. Transthoracic echocardiography revealed normal ventricular systolic function.

**Figure 1:**
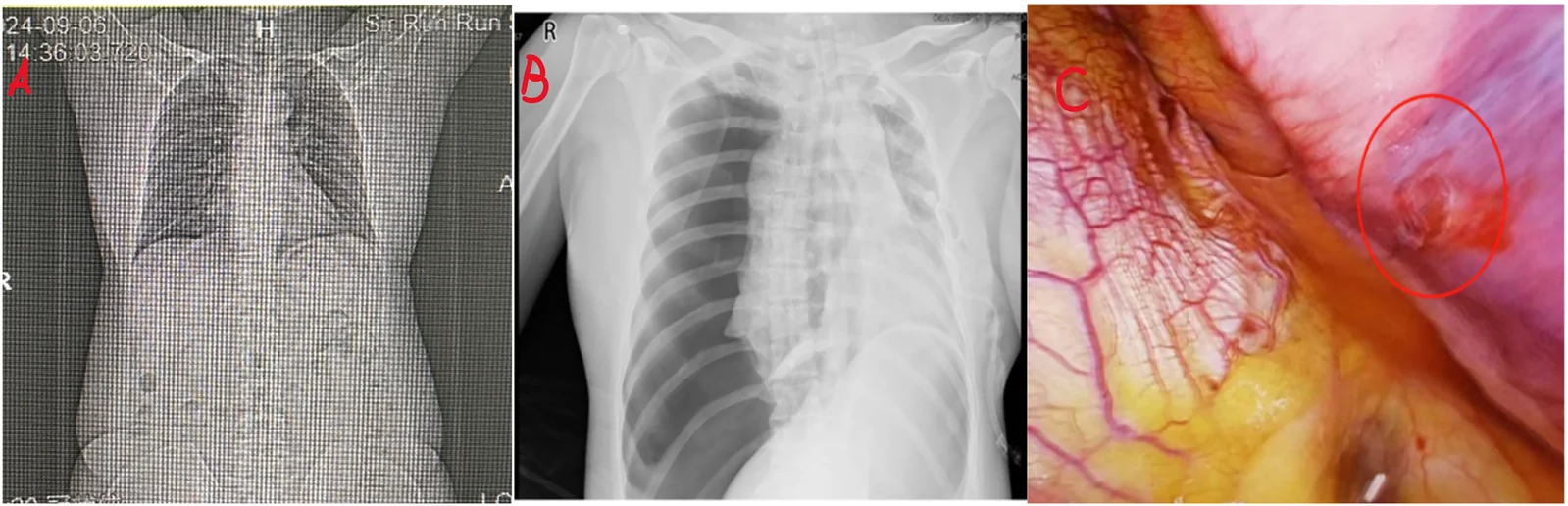
**(A)** chest CT reveals multiple ground-glass opacities in both lungs. The largest lesion is located in the left upper lobe, measuring approximately 6 × 7 mm. A small solid nodule is present in the right upper lobe, and calcification is noted in the left oblique fissure. Minimal fibrotic foci are observed in both lungs. Slight thickening of the upper pleura bilaterally. **(B)** A bedside lateral chest radiography revealed 90% compression of the right lung. **(C)** Thoracoscopic examination revealed partial adhesions between the right lung and the chest wall. A localized band of adhesions at the base of the right lower lobe was found to be partially ruptured. No pleural effusion was observed, and no chest wall nodules were detected.

On arrival in the operating room, blood pressure (BP) was 140/82 mmHg, heart rate (HR) 83 beats per min(bpm), and peripheral oxygen saturation (SpO₂) 93% on room air, increasing to 98% after 3 min of preoxygenation with 100% oxygen. General anesthesia was induced with remimazolam 24 mg, rocuronium 60 mg, and sufentanil 30 μg. Tracheal intubation was performed using a video laryngoscope. The glottic opening was clearly visualized, and a 37-Fr left-sided DLT was advanced smoothly through the vocal cords without significant resistance. The tube was successfully positioned on the first attempt, with no obvious airway bleeding or mucosal injury. Bilateral breath sounds were present on auscultation. Appropriate lung isolation was confirmed before one-lung ventilation (OLV). Fiberoptic bronchoscopy subsequently confirmed appropriate positioning of the bronchial lumen within the left main bronchus, and the tube was secured at 28.5 cm from the incisors. Volume-controlled ventilation was then initiated, with the parameters for two-lung ventilation (TLV) set as shown in Table 1.

**Table 1:** Intraoperative ventilatory management.

Ventilatory stage	VCV to lung(s)	VT (mL)	RR (breaths/min)	FiO₂	PEEP (cmH₂O)	CPAP to operative lung	PIP (cmH₂O)	Pplat (cmH₂O)	SpO₂
Initial TLV	Both lungs	450	12	100%	0	-	15	13	100%
Initial OLV	Right lung	350	16	100%	0	-	15	13	85%
Rescue ventilation during hypoxemia	Right lung	400	16	100%	5	Left lung: 5 cmH₂O, O₂ 5 L/min for 10 min	15–20	13–18	100%
Stabilized OLV	Right lung	400	16	40–50%	5	-	15–20	13–18	97–100%

**Table 2:** Timeline of clinical changes and management after extubation.

Time Point	Vital Signs	Clinical Status/Findings	management
T0(at extubation)	SpO₂ 99%, HR 98 bpm, RR 18 breaths/min, ABP 143/79 mmHg	conscious (Ramsay score 2), compliance with instructions to look up > 10 s, spontaneous VT 300–400 mL, intermittent vigorous coughing(PIP elevations to 30–40 cmH₂O)	DLT extubation; oxygen via nasal cannula at 3 L/min
T + 3 min	SpO₂ 85%, HR 110 bpm, RR 30 breaths/min; ABP 125/69 mmHg	dyspnea, cyanosis of the lips, restlessness	oxygen switched to face mask at 8 L/min; anesthesiologist notified
T + 5 min	SpO₂ 79%, HR 111 bpm，RR 28 breaths/min, ABP 128/75mmHg	high airway pressure when squeezes	open the airway, bag-valve-mask ventilation was initiated (100% inspired oxygen, 10 L/min); physical examination
T + 7 min	SpO₂ 80%, HR 115 bpm, RR 26 breaths/min, ABP 124/75 mmHg	physical examination revealed no obvious subcutaneous emphysema, bilateral symmetrical thoracic cage, and scattere mild wheezing in both lungs, continuous air bubbling from the left chest tube	salbutamol (200 μg, two puffs); methylprednisolone (40 mg, IV); propofol (50 mg, IV)
T + 10 min	SpO₂ 82%, HR 112 bpm, RR 28 breaths/min, ABP 122/75 mmHg, RR 12 bpm	persistent wheezing in both lungs	propofol (100 mg, IV); succinylcholine (100 mg, IV); emergency re-intubation performed; ventilator in VCV mode (FiO₂ 100%, VT 450 ml, RR 12 bpm, PEEP 3cmH_2_O, I:E 1:2;
T + 13 min	SpO₂ 88%, HR 115 bpm, RR 17 breaths/min, ABP 108/60 mmHg	PIP alarms at 35 cm H₂O; auscultation reveals wheezing in the left lung and diminished breath sounds in the right lung	methylprednisolone (40 mg, IV); fiberoptic bronchoscopy (ruled out tube malposition/obstruction); VT decreased to 360 ml, RR increased to 17 bpm.
T + 15 min	SpO₂ 83%, HR 118 bpm, RR 17 breaths/min, ABP 79/56 mmHg	PIP 40 cmH₂O.	call the lead surgeon and a senior anesthesiologist; metaraminol (0.5 mg, IV), salbutamol (200 μg, two puffs, via the endotracheal tube); accelerate fluid replacement
T + 17 min	SpO₂ 75%, HR 120 bpm; RR 17 breaths/min, ABP 63/44 mmHg	marked cyanosis, persistent airway hypertension	norepinephrine (20*μ*g, IV); arterial Blood Gas Analysis
T + 20 min	SpO₂ 77%, HR 120 bpm; RR 17 breaths/min, ABP 96/63 mmHg	bulging of the right hemithorax, absent breath sounds on the right, clear breath sounds on the left, arterial blood gas analysis: PH:7.374, PO_2_: 159mmHg, PCO_2_: 35.6 mmHg, BE:3.9, K^+:^3.6 mmol/L, CA^2+:^1.05 mmol/L	emergency bedside chest x-ray requested; proper endotracheal tube position, marked narrowing of the right middle and lower lobe bronchi confirmed by bronchoscopy
T + 30 min	SpO₂ 80%, HR 118 bpm; RR 17 breaths/min, ABP 95/60 mmHg	chest radiography: a massive right-sided pneumothorax with near-complete collapse of the right lung and leftward mediastinal shift, with associated leftward deviation of the trachea	emergency needle decompression (the second intercostal space corresponds to the midclavicular line)
T + 32 min	SpO₂ 91%, HR 108 bpm; RR 17 breaths/min, ABP 118/83 mmHg	PIP 20 cmH₂O.	followed by right chest tube insertion, ventilator settings adjusted (FiO₂ 50%,VT 450 mL, RR 13 bpm); insert a closed-chest drainage tube into the thoracic cavity and connect it to negative pressure at 10 cmH_2_O
T + 35 min	SpO₂ 96%, HR 90–100 bpm; RR 13 breaths/min, ABP 118/83 mmHg	rosy complexion	patient stabilized and returned to surgical repair

After inserting the thoracoscope into the left pleural cavity, OLV was initiated (see Table 1 for parameters), and SPO₂ gradually decreased to 85%. To improve oxygenation, the tidal volume (VT) was increased to 400 mL, and positive end-expiratory pressure (PEEP) of 5 cmH₂O was applied to the right ventilated lung. Concurrently, continuous positive airway pressure (CPAP) of 5 cmH₂O was administered to the left operative lung at an oxygen flow rate of 5 L/min for approximately 10 min. Following these interventions, SpO₂ rose to 100%. Once oxygenation stabilized, fraction of inspired oxygen (FiO₂) was gradually reduced to 50%. After 30 min of one-lung ventilation, arterial blood gas analysis showed PaO₂ of 79 mmHg, PaCO₂ of 47 mmHg, and SpO₂ of 95%. One-lung ventilation continued until the intrathoracic surgery was completed, for a total of 45 min. During stabilized one-lung ventilation, end-tidal carbon dioxide (EtCO₂) remained between 33 and 45 mmHg. Peak inspiratory pressure (PIP) ranged from 15 to 20 cmH₂O, while plateau pressure (Pplat) ranged from 13 to 18 cmH₂O. With a PEEP of 5 cmH₂O, the corresponding driving pressure ranged from approximately 8 to 13 cmH₂O. Prior to closing the thoracic cavity, the airway was suctioned. Subsequently, under direct surgical visualization, the collapsed left lung was slowly re-expanded using an airway pressure of 25 cmH₂O until uniform expansion of all lung lobes was observed. The double-lumen bronchial tube was then withdrawn appropriately to a position 22 cm from the incisors, secured in place, and bilateral lung ventilation was restored. At the end of the approximately 50-min procedure, a closed thoracic drainage tube was placed in the left pleural cavity.

Since the patient has not yet regained consciousness, she was transferred to PACU in accordance with our hospital's procedures to continue mechanical ventilation. Mechanical ventilation was continued in volume-controlled synchronized intermittent mandatory ventilation (VC-SIMV) mode, with VT 450 ml, FiO₂ 30%, RR 12 breaths/min, and PEEP 5cmH_2_O. The chest tube was securely in place with unobstructed drainage, and no significant subcutaneous emphysema was observed. The patient did not respond to verbal stimulation. Five minutes after administration of sugammadex sodium(50 mg) injection, the patient was assessed as alert (Ramsay scale score of 2). During recovery, she experienced repeated episodes of vigorous coughing. Her spontaneous tidal volume was 300–400 mL, and she was able to sustain a head lift for 10 s on command. As she met the institutional criteria for extubation, the DLT was removed. Oxygen was then administered via nasal cannula at 3 L/min, and SpO₂ was initially maintained at 99%.

After extubation, the patient developed acute hypoxemia, tachypnea, and bilateral wheezing, considering the possibility of bronchospasm. However, her condition deteriorated despite maximal medical therapy, including intravenous methylprednisolone, nebulized salbutamol, and deep sedation with propofol. Even after re-intubation, hypotension and ventilation difficulties occurred. Twenty minutes after extubation, physical examination revealed a bulging right thorax with absent breath sounds on the right, prompting an emergent chest radiography. The radiograph confirmed a right-sided tension pneumothorax with near-complete lung collapse (Figure 1B). Immediate needle decompression and subsequent chest tube insertion resulted in rapid clinical improvement. The specific clinical dynamic changes and rescue process are detailed in Table2. The patient returned to the operating room for thoracoscopy. Partial adhesions were observed between the right lung and the chest wall. No pleural effusion was present. A localized rupture of the adhesion band in the basal segment of the right lower lobe was identified, accompanied by lung tissue damage and air leakage (Figure 1C).

Surgical repair was performed successfully. After surgery, the patient remained intubated until she was fully awake and demonstrated adequate spontaneous ventilation, stable oxygenation, and stable hemodynamics. Tracheal extubation was then performed without complication. She recovered uneventfully thereafter. Follow-up imaging showed complete resolution of the pneumothorax without recurrence. She was discharged on postoperative day 7 and remained asymptomatic at the 1-month follow-up.

## 3 Discussion

Contralateral pneumothorax is a rare but recognized complication after thoracoscopic surgery, with a reported incidence of approximately 0.16% (7). Its clinical manifestations (7–9), including hypoxemia, dyspnea, wheezing, and hemodynamic instability, can be difficult to distinguish from common postoperative complications such as atelectasis and bronchospasm, particularly in the PACU. In this case, contralateral pneumothorax developed after extubation and initially presented with nonspecific respiratory abnormalities. Therefore, unexplained or worsening respiratory abnormalities after extubation, especially in patients who received one-lung ventilation, should raise suspicion of contralateral pneumothorax.

The second surgery confirmed a rupture in a right pleural adhesion band with persistent air leakage, providing direct anatomical evidence for the development of pneumothorax. Combined with the mild bilateral pulmonary fibrosis and pleural thickening seen on preoperative CT, these findings suggest that pre-existing pleuropulmonary structural abnormalities may have created a local tissue vulnerability. Positive-pressure ventilation during one-lung ventilation may have further increased local mechanical stress. Previous studies have shown that higher tidal volumes and prolonged one-lung ventilation can increase mechanical stress and the risk of postoperative pulmonary complications (10). In this case, the tidal volume during one-lung ventilation reached approximately 7 mL/kg predicted body weight (PBW), exceeding the commonly recommended range of 4–6 mL/kg PBW for lung-protective one-lung ventilation. Although PIP, Pplat, and driving pressure did not show sustained elevations, the relatively high tidal volume may nevertheless have contributed to cumulative local mechanical stress in the presence of pre-existing pleuropulmonary abnormalities.

In contrast, the temporal association between repeated vigorous coughing during emergence and the development of pneumothorax was more apparent. Vigorous coughing can cause a rapid increase in intrathoracic pressure and abrupt displacement of lung tissue. In this case, PIP transiently increased to approximately 30 cmH₂O during coughing, indicating substantial transient airway pressure fluctuations. In patients with pleural adhesions or localized fibrotic changes, such abrupt pressure changes and mechanical traction may concentrate stress at adhesion sites, potentially leading to rupture of the adhesion band or adjacent lung tissue (4, 11, 12). Thus, vigorous coughing during extubation may be more than a transient emergence-related response in patients with pleuropulmonary structural abnormalities and may act as an important mechanical trigger for lung injury. This highlights the potential importance of cough control during the peri-extubation period.

Several strategies have been proposed to reduce vigorous coughing and associated airway pressure fluctuations during extubation, including adequate analgesia, gradual cuff deflation, and the administration of lidocaine or dexmedetomidine (13, 14). Minimizing unnecessary airway stimulation, including appropriate selection of DLT size and limiting repeated airway suctioning, may further reduce airway irritation during emergence. In the present case, although the patient had no history of smoking, airway inflammation was present, and a DLT was used for one-lung ventilation. Despite the use of topical lidocaine spray, local anesthetic infiltration at the incision site, and intravenous dexmedetomidine to attenuate coughing, vigorous coughing still occurred during emergence. Compared with a single-lumen endotracheal tube, a DLT is larger and relatively rigid, with its distal portion extending through the trachea, across the carina, and into the main bronchus, potentially resulting in greater mechanical stimulation of the airway and a more pronounced cough response during emergence.

Previous studies have explored replacing the DLT with a supraglottic airway device or single-lumen endotracheal tube before emergence to reduce airway irritation and cough-related complications (2). However, airway exchange itself carries risks. A simulation study found that only 37% of anesthesiologists could complete the exchange within 10 min when the endotracheal tube was partially obstructed, and complications such as airway loss and severe hypoxemia could occur (15). Therefore, pre-extubation airway exchange should be individualized according to the patient's airway condition, oxygenation reserve, surgical procedure, and the experience of the anesthesia team, rather than adopted as a routine strategy.

After extubation, the patient initially developed bilateral wheezing, progressive hypoxemia, and increased airway pressure, all of which can occur with bronchospasm, consistent with previous reports (16). Thus, considering bronchospasm as the initial diagnosis was clinically reasonable. However, on reassessment approximately 13 min after extubation, breath sounds on the right side were markedly diminished, which was an important warning sign of right-sided pneumothorax. Nevertheless, the initial diagnostic framework and persistent wheezing on the left may have led the PACU team to prioritize more common perioperative respiratory complications, such as bronchospasm and tube malposition.

This diagnostic challenge can be understood from the perspective of cognitive bias. Early literature has described the failure to sufficiently reconsider an initial diagnostic hypothesis when new information emerges as “fixation error” (17, 18). Similar diagnostic difficulties have been reported in thoracic anesthesia, where hypoxemia, increased airway pressure, and hypotension, although important warning signs of tension pneumothorax, are also relatively common during one-lung ventilation and thoracic surgery and may therefore have limited diagnostic specificity. The challenge, therefore, may not be the absence of abnormal signs, but rather that these signs can be incorporated into an existing perioperative explanation. This case highlights the importance of reassessing the initial diagnosis when new findings cannot be fully explained by the existing diagnostic framework. Point-of-care lung ultrasound can rapidly identify signs of pneumothorax, such as absent lung sliding and the lung point, and may be particularly valuable in hemodynamically unstable patients (6, 19). When tension pneumothorax is strongly suspected and the patient is rapidly deteriorating, pleural decompression and definitive treatment should not be delayed solely to obtain confirmatory imaging.

From a respiratory mechanics perspective, bronchospasm and tension pneumothorax may present with similar clinical findings but have different underlying mechanisms. Bronchospasm primarily increases airway resistance, typically causing a marked increase in PIP with relatively preserved or only mildly increased Pplat, resulting in a larger PIP–Pplat difference. In contrast, as pleural pressure increases and the affected lung becomes compressed, tension pneumothorax can reduce respiratory system compliance, leading to parallel increases in both PIP (20). A limitation of this case is that PIP, Pplat, and their difference were not continuously recorded during clinical deterioration. Therefore, dynamic changes in respiratory mechanics could not be used to further distinguish increased airway resistance from reduced respiratory system compliance.

In response to the challenges in early recognition identified in this case, we subsequently strengthened cough management during extubation in the PACU, provided training in the differential diagnosis of postoperative respiratory complications, including tension pneumothorax, atelectasis, bronchospasm, and airway secretion obstruction, and introduced lung ultrasound training to improve the bedside recognition of intrathoracic abnormalities such as pneumothorax.

## 4 Conclusion

In summary, this case highlights an extremely rare instance of contralateral TP following thoracoscopic surgery, which occurred immediately after extubation and presented with clinical features similar to those of bronchospasm. In patients undergoing one-lung ventilation, pre-existing pleuropulmonary abnormalities and perioperative positive-pressure ventilation may further increase susceptibility to mechanical lung injury. Vigorous coughing during extubation can cause abrupt increases in intrathoracic and airway pressures and may serve as an additional mechanical trigger. When postoperative respiratory deterioration does not respond well to standard treatment for bronchospasm, TP should always be considered as a key differential diagnosis. Early use of bedside lung ultrasound aids in the timely identification and management of this life-threatening complication.

## Data Availability

The original contributions presented in the study are included in the article/Supplementary Material, further inquiries can be directed to the corresponding author.

## References

[1] Chen Z Dong Q Liang L. Effect of different thoracic anesthesia on postoperative cough. J Thorac Dis. (2018) 10:3539–47. 10.21037/jtd.2018.05.126 30069351 PMC6051848

[2] Ishibashi H Wakejima R Kurihara Y Seto K Baba S Asakawa A. Extubation by supraglottic airway after lobectomy prevents cough-associated air leaks and prolonged coughing or sore throat. Eur J Cardiothorac Surg. (2023) 63:ezad158. 10.1093/ejcts/ezad158 37079745

[3] Irwin RS Dudiki N French CL Abu Dabrh AM Altman KW Azoulay E. Life-threatening and non-life-threatening complications associated with coughing. Chest. (2020) 158:2058–73. 10.1016/j.chest.2020.06.012 32565267 PMC8640838

[4] Yoon JS Choi SY Suh JH Jeong JY Lee BY Park YG. Tension pneumothorax, is it a really life-threatening condition? J Cardiothorac Surg. (2013) 8:197. 10.1186/1749-8090-8-197 24128176 PMC4016536

[5] Huan NC Sidhu C, TR pneumothorax. Clin Chest Med. (2021) 42:711–27. 10.1016/j.ccm.2021.08.007 34774177

[6] Jalota Sahota R Sayad E. Tension pneumothorax. In: *StatPearls*. Treasure Island (FL): StatPearls Publishing (2026). Available online at: http://www.ncbi.nlm.nih.gov/books/NBK559090/. (Accessed July 28, 2026).

[7] Chang AH Chen H Li L Hu Y Zhang R Zhang X. Contralateral tension pneumothorax in one-lung ventilation: a case report and systematic review. Cureus. (2024) 16:e61306. 10.7759/cureus.61306 38813077 PMC11135384

[8] Ki S Choi B Cho SB Hwang S Lee J. Unexpected tension pneumothorax developed during anesthetic induction aggravated by positive pressure ventilation: a case report. Medicina (B Aires). (2023) 59:1631. 10.3390/medicina59091631 PMC10535224 37763751

[9] Kumata S Matsuoka K Nagai S Ueda M Okada Y Miyamoto Y. Contralateral tension pneumothorax during video-assisted thoracic surgery for lung cancer in a patient with obesity and rib fractures: a case report and review of the literature. J Med Case Rep. (2020) 14:221. 10.1186/s13256-020-02556-w 33187545 PMC7666458

[10] So MC Machado de Souza C Buduhan G Srinathan SK Tan L Kidane B. Variation in and deviation from protective ventilation during lung surgery. JTCVS Tech. (2025) 35:102134. 10.1016/j.xjtc.2025.10.010 41658871 PMC12881778

[11] Hsu CW Sun SF. Iatrogenic pneumothorax related to mechanical ventilation. World J Crit Care Med. (2014) 3:8–14. 10.5492/wjccm.v3.i1.8 24834397 PMC4021154

[12] Sato R Arita M Takahashi H Amano A Tanaka A Hamakawa M. Risk factors for the onset of pneumothorax in idiopathic pulmonary fibrosis. Respir Investig. (2025) 63:744–48. 10.1016/j.resinv.2025.06.007 40543434

[13] Purohit A Kumar M Kumar N Bindra A Pathak S Yadav A. Comparison between dexmedetomidine and lidocaine for attenuation of cough response during tracheal extubation: a systematic review and meta-analysis. Indian J Anaesth. (2024) 68:415–25. 10.4103/ija.ija_790_23 38764958 PMC11100647

[14] Wang X Zhu G Tan J Cao X Duan M. Effects of continuous and slow tracheal tube cuff deflation on cough reflex during extubation in noncardiac surgery patients: a randomised clinical trial. BMC Anesthesiol. (2023) 23:76. 10.1186/s12871-023-02003-5 PMC9903553 36750765

[15] Kriege M Demare T Ruemmler R Schmidtmann I Wojciechowski J Busch A. Exchange of a tracheal tube and supraglottic airway device: evaluation of different techniques in three simulated airway scenarios (TUBE study)—a prospective, randomised controlled study. J Clin Med. (2024) 13:16. 10.3390/jcm13010016 PMC10779719 38202022

[16] Mohd Fauzi NA Mohamad I Sheikh Ab Hamid S. Tension pneumothorax with cardiac arrest complicating bronchoscopy in a nut-aspirated child. Malays J Paediatr Child Health. (2022) 28:70–3. 10.51407/mjpch.v28i1.157

[17] Fioratou E Flin R Glavin R. No simple fix for fixation errors: cognitive processes and their clinical applications. Anaesthesia. (2010) 65:61–9. 10.1111/j.1365-2044.2009.05994.x 20121773

[18] Schmidt HG Mamede S Van Den Berge K Van Gog T Van Saase J Rikers RMJP. Diagnostic errors induced by a wrong *a priori* diagnosis: a prospective randomized simulator-based trial. BMJ Qual Saf. (2020) 29:967–75.

[19] Metzler EK Everløff N Juul AD Laursen CB Graumann O Pietersen PI. Value of focused lung ultrasound in diagnosing and monitoring pneumothorax after CT-guided lung biopsy. J Ultrasound. (2025) 28:11–8. 10.1007/s40477-025-00998-w 40014278 PMC11947390

[20] Flynn BC Miranda HG Mittel AM Moitra VK. Stepwise ventilator waveform assessment to diagnose pulmonary pathophysiology. Anesthesiology. (2022) 137:85–92. 10.1097/ALN.0000000000004220 35511174

